# Novel Hybrid Composites Based on PVA/SeTiO_2_ Nanoparticles and Natural Hydroxyapatite for Orthopedic Applications: Correlations between Structural, Morphological and Biocompatibility Properties

**DOI:** 10.3390/ma13092077

**Published:** 2020-05-01

**Authors:** Simona Cavalu, Luminita Fritea, Marcel Brocks, Katia Barbaro, Gelu Murvai, Traian Octavian Costea, Iulian Antoniac, Claudio Verona, Martina Romani, Alessandro Latini, Romano Zilli, Julietta V. Rau

**Affiliations:** 1 Faculty of Medicine and Pharmacy, University of Oradea, P-ta 1 Decembrie 10, 410081 Oradea, Romania; fritea_luminita@yahoo.com; 2 Biomedical Sciences Doctoral School, Faculty of Medicine and Pharmacy, University of Oradea, P-ta 1 Decembrie 10, 410081 Oradea, Romania; physioempathie@gmail.com (M.B.); gelu_murvai@yahoo.com (G.M.); 3 Istituto Zooprofilattico Sperimentale del Lazio e della Toscana “M. Aleandri”, Via Appia Nuova 1411, 00178 Rome, Italy; katia.barbaro@izslt.it (K.B.); romano.zilli@izslt.it (R.Z.); 4 Advanced Materials Research Laboratory, University of Oradea, 1 Universitatii Street, 410087 Oradea, Romania; costea.traian.octavian@gmail.com; 5 Department Materials Science and Engineering, University Politehnica of Bucharest, Splaiul Independentei 313, sector 6, 060032 Bucharest, Romania; antoniac.iulian@gmail.com; 6 Department of Industrial Engineering, University “Tor Vergata” Rome, via del Politecnico 1, 00133 Rome, Italy; claudio.verona@uniroma2.it; 7 INFN National Laboratory of Frascati, via Enrico Fermi 40, 00044 Frascati, Italy; martina.romani@lnf.infn.it; 8 Department of Chemistry, Sapienza University of Rome, Piazzale Aldo Moro 5, 00185 Rome, Italy; alessandro.latini@uniroma1.it; 9 Istituto di Struttura della Materia, Consiglio Nazionale delle Ricerche (ISM-CNR), Via del Fosso del Cavaliere 100, 00133 Rome, Italy; giulietta.rau@ism.cnr.it

**Keywords:** PVA (poly(vinyl alcohol)), composites, hydroxyapatite, selenium, titania, nanoparticles, BMMSC differentiation

## Abstract

The properties of poly(vinyl alcohol) (PVA)-based composites recommend this material as a good candidate for the replacement of damaged cartilage, subchondral bone, meniscus, humeral joint and other orthopedic applications. The manufacturing process can be manipulated to generate the desired biomechanical properties. However, the main shortcomings of PVA hydrogels are related to poor strength and bioactivity. To overcome this situation, reinforcing elements are added to the PVA matrix. The aim of our work was to develop and characterize a novel composition based on PVA reinforced with Se-doped TiO_2_ nanoparticles and natural hydroxyapatite (HA), for possible orthopedic applications. The PVA/Se-doped TiO_2_ composites with and without HA were structurally investigated by FTIR and XRD, in order to confirm the incorporation of the inorganic phase in the polymeric structure, and by SEM and XRF, to evidence the ultrastructural details and dispersion of nanoparticles in the PVA matrix. Both the mechanical and structural properties of the composites demonstrated a synergic reinforcing effect of HA and Se-doped TiO_2_ nanoparticles. Moreover, the tailorable properties of the composites were proved by the viability and differentiation potential of the bone marrow mesenchymal stem cells (BMMSC) to osteogenic, chondrogenic and adipogenic lineages. The novel hybrid PVA composites show suitable structural, mechanical and biological features to be considered as a promising biomaterial for articular cartilage and subchondral bone repair.

## 1. Introduction

PVA (poly(vinyl alcohol))-based materials are considered as the most promising synthetic biomaterials to be used in various pharmaceutical, cosmetic or biomedical applications, like drug delivery, tissue engineering, tissue regeneration or replacement [1,2]. PVA hydrogels and membranes have been developed years ago for various medical purposes: bone and cartilage repair [3,4], contact lenses [5,6], hemodialysis membranes [7], tissue regeneration scaffolds [8,9,10,11,12,13]. So, the versatility of PVA is an important advantage for the formulation of new multifunctional biomaterials to be applied in the biomedical field [8]. PVA’s properties, either alone or in combination with inorganic particles, make it a good biomaterial candidate for simulating natural tissues, such as bone, cartilage and meniscus, with suitable elastic and compressive mechanical properties, as the manufacturing process can be manipulated to generate the desired biomechanical properties [9]. In order to form hydrogels for medical applications, PVA needs to be crosslinked either physically or chemically, providing structural stability upon the swelling process of water or different biological fluids. Chemical crosslinking can be performed using chemicals, such as glutaraldehyde, maleic acid, citric acid, propranolol or acetone [10], whereas the physical crosslinking methods are based on air-drying and cryogelation by repeated freeze-thaw cycles of the aqueous polymer solutions [11]. It has been demonstrated that many of the chemicals used for chemical crosslinking are not cytocompatible [12], and hence, a physical method is often preferred not only because eliminating the need of potential toxic cross-linking agents, but also because the physically cross-linked PVA gels display higher mechanical strength and stiffness due to the presence of microcrystals as opposite to the chemically cross-linked counterparts [13,14,15]. On the other hand, the poor bioactivity and mechanical resistance of PVA hydrogels, along with the lack of adhesive and mechanical fixation to the natural tissue, could be improved by adding hydroxyapatite (HA) as the reinforced phase to the PVA composite, providing active sites both for biomineralization and for cellular attachment. The development of biocomposite materials has been always motivated by the fact that pure materials alone cannot meet all requirements for biomedical implants. Combining polymer with inorganic material is one of the most promising strategies to enhance mechanical and bioactivity properties of polymers, the resulting composites being more stable in terms of thermo-mechanical features in conjunction with good biocompatibility [16]. Titanium dioxide (TiO_2_) is also considered to be one of the best choices as inorganic filler due to its outstanding biocompatibility and biomechanical properties. It is the most commonly used inorganic filler in orthopedic prostheses due to its excellent corrosion resistance and biocompatibility. Besides the corrosion resistance and thermo-mechanical stability, the advantages of TiO_2_ in implant and tissue engineering applications are related to the formation of HA via the Ti-OH site and the ability to accelerate bone growth [2]. Another advantage is the antibacterial activity of TiO_2_ in the form of nanoparticles, which demonstrated better inhibition of bacterial growth, with broad-spectrum antimicrobial action [2,17]. It has been demonstrated that TiO_2_ nanoparticles have higher bioactivity than conventional (micron) particle sizes. For example, when exposed to nanophase TiO_2_ particles, osteoblasts and chondrocytes show a well-spread morphology and increased proliferation, compared with cells exposed to particles of conventional size [18]. Up to now, different nanofiller types were investigated in order to tailor the properties of PVA hydrogels for biomedical applications: ZrO_2_ [19], clays [20], SiO_2_ [21], TiO_2_ [22], Al_2_O_3_ [23], Ag [24], nano-HA [25] and graphene [26]. The challenge in developing orthopedic applications of PVA is still actual, even if the first reported paper dealing with the use of PVA for articular cartilage repair was mentioned in the early 1970s [27]. The PVA-based composites, as a substitute material for the bone and/or cartilage, needs to bear dynamic loading in the joint movement besides the static load of joint contact. So, in the current context, the aim of our work was to develop and characterize a novel composition based on PVA reinforced with natural HA and Se-doped TiO_2_ nanoparticles, for possible applications as bone and/or cartilage replacement. Selenium nanoparticles were also chosen for this study, because of their antimicrobial effect and chemo-preventive properties, by immunological stimulation [28]. For this purpose, structural and morphological analyses were applied using FTIR and XRF spectroscopies, XRD analysis and SEM investigation. Nano-mechanical properties were emphasized by nanoindentation measurement, while the biological behavior of the composites was assessed in vitro, using bone marrow mesenchymal stem cells (BMMSC), in terms of cells viability, differentiation and proliferation.

## 2. Materials and Methods

### 2.1. Fabrication and Structural Characterization of Se-Doped TiO_2_ Nanoparticles

Sodium hydrogen biselenite (NaHSeO_3_, 96%, MW 150.96 g/mol, (Alfa Aesar, Kandel, Germany) and titania powder (TiO_2_, MW 79.86 g/mol, Sigma-Aldrich, Steinheim, Germany) were used to prepare Se-doped TiO_2_ nanoparticles, following the steps: (1) 6 g TiO_2_ powder was dissolved in NaOH, 10 M, by continuous stirring at 30 °C, for 2 h. (2) Autoclave treatment was applied at 140 °C for 24 h. (3) Removing the supernatant, the TiO_2_ particles were washed with a solution of HCl 0.1 M, centrifuged at 6000 r/min for 5 min, washed again with ultrapure water and dried at 80 °C for 18 h. (4) The calcination of TiO_2_ particles was performed in a standard furnace (Nabertherm GmbH, Lilienthal, Germany) at different temperatures: 400 °C, 600 °C and 800 °C for 2 h. (5) Each set of resulted particles was allowed to react at 100 °C with a mixture of NaHSeO_3_ and lactose monohydrate (VWR Chemicals, Vienna, Austria) in a molar ratio of 1:3. The ratio NaHSeO_3_/TiO_2_ was 1/6 (w/w). The characteristic red color, as an indicator of Se nanoparticles formation, was reached after heating the mixture at 100 °C, for 30 min [28,29,30]. Finally, the resulting particles were washed three times with deionized water, filtered and dried at room temperature. The samples were named SeTi400, SeTi600 and SeTi800, according to the calcination temperature of the TiO_2_ starting powder. In order to evaluate the morphological features and the crystallinity, the powder samples were investigated by SEM analysis (Leo 438VP Scanning Electron Microscope, Oberkochen, Germany) and XRD (X-ray diffractometer MiniFlex 600, Rigaku, Tokyo, Japan) operating at 40 kV, 15 mA, with CuKα monochromatic radiation.

### 2.2. Fabrication of PVA/HA/Se-Doped TiO_2_ Composites

PVA (98% hydrolyzed, MW 72,000 g/mol) was purchased from Merck Chemical Co (Darmstadt, Germany). HA powder was biologically derived from bovine bone by calcination method as described in our previous work [30] obtaining a quasi-homogeneous particle size distribution, with an average size of 1.8 μm. Two sets of PVA composites were prepared, with and without HA. For the first set, PVA aqueous solution in a concentration of 10%, was prepared by dissolving weighed PVA in deionized water and then heating it at 90 °C for 2 h with continuous stirring. Se-doped TiO_2_ powder was dissolved in distilled water, in a concentration of 2.5% (w/v), sonicated and then added to the PVA solution by continuous stirring until a good dispersion was achieved. The reference PVA solution (10%) and PVA-containing Se-doped TiO_2_ particles were poured in Petri dishes and allowed to dry overnight. The first set of samples was named PVA, PVA400, PVA600 and PVA800. For the second set, the same procedure was applied, but in the final stage, the natural HA powder (dispersed in water) was added to each PVA/Se-doped TiO_2_ mixture, in a concentration of 10% (w/v), magnetically stirred at room temperature and finally transferred into Petri dishes. The second set of samples was named PVAHA; PVA400HA, PVA600HA and PVA800HA. Three freeze-thaw cycles were applied to the specimens: each Petri dish was frozen at −20 °C for 6 h in a low-temperature freezer, then taken out and thawed for 6 h at room temperature (25 °C) in preparation for the next freezing process. 

### 2.3. Structural Characterization of PVA/HA/Se-TiO_2_ Composites

FTIR spectroscopic measurements in the ATR mode were performed by using a Tensor II spectrometer (Bruker Optics, Ettlingen, Germany) equipped with a diamond reflection element (Platinum ATR accessory) and a DTGS detector. Measurements were collected in the range of 4500−400 cm^−1^ at a 4 cm^−1^ resolution, using 32 scans. The XRF spectra were obtained by using a portable spectrometer ELIO by XGLab (Ettlingen, Germany), with an energy resolution of <135 eV on the MnKα. In this work, the following measurement conditions were used: 40 keV tube voltage, 80 μA tube current and 60 s acquisition time. XRD measurements were carried out with a Malvern Panalytical X’Pert Pro MPD diffractometer in Bragg-Brentano configuration equipped with a Cu Kα source (λ = 1.54184 Å) and an ultrafast RTMS X’Celerator detector (Malvern, UK). Morphological characterization of the composites was performed using a Leo 438VP electron microscope (SEM, Oberkochen, Germany), operating at 30 kV, with a variable vacuum level, equipped with an EDX (Energy Dispersive X-ray Spectroscopy, Oxford Instruments, Wiesbaden, Germany) unit, obtaining cross-section images of the PVA composites with different details. PVA specimens with and without HA were subjected to nanoindentation tests using a depth-sensing Nanoindenter G200 device (Keysight Agilent Technologies, Santa Clara, CA, USA), at room temperature and normal humidity. A pyramidal shaped tip (Berkovich) was used to assess the mechanical properties. During the nanoindentation test, the tip penetrates into the sample surface by applying a load, each indentation consisting of a loading and unloading phase. In order to obtain accurate results, each nanoindentation test was repeated 16 times, while the optical image of the sample using a microscope allowed precise control between the sample position and the indenter. Young modulus values were obtained from load–displacement curves, by fitting parameters, using the Oliver–Pharr method [31,32].

### 2.4. In Vitro Cells Culture

Stem cells were isolated from the bone marrow of a 2-year-old horse (BMMSC), added with anticoagulant (sodium citrate), diluted 1:3 with a sterile physiological solution (0.9% (w/v) NaCl) and stratified on Ficoll-Paque TM Plus GE Healthcare (Sigma-Aldrich). After centrifugation at 1500× *g* for 10 min at room temperature, the ring of mononuclear cells was taken and transferred to another test tube, diluted with αMEM (Gibco, Waltham, MA, USA) and centrifuged again at 250× *g* for 10 min at room temperature without brake. Finally, the cells were resuspended in growth medium αMEM (Gibco) supplemented with FBS (fetal bovine serum, Gibco) at 10%, 100 U/mL penicillin (Gibco), 100 µg/mL of streptomycin (Gibco) and 8 µg/mL fungizone (Gibco) and transferred to the flask cell cultures and incubated at 37 °C with 5% CO_2_. The growth medium was changed every three days. As the cells reached the confluence of 75–80%, they were transferred from one flask to another, using trypsin (Gibco). The morphological features were inspected every day by an inverted optical microscope (NIKON Eclipse TE2000-U, Melville, NY, USA).

#### 2.4.1. Differentiation in the Different Lineages

The differentiation potentials of BMMSCs in the osteogenic, adipogenic and chondrogenic lineages have been evaluated according to the protocol described by Rau et al. [33]. The cells were enzymatically detached and placed in 24-well plates at concentrations of 40.000 cells/mL in αMEM medium and incubated at 37 °C with 5% CO_2_ for 24 h. The stimulation with the appropriate means of differentiation was performed according to the following conditions (the negative control being the cells grown in αMEM containing 10% FBS):

(1) Osteogenic differentiation: 3 weeks of stimulation in αMEM containing 10% FBS, ascorbic acid 50 µg/mL, ß-glycerophosphate 10 mM and dexamethasone 10^−7^ M, the negative control being the same cells grown in αMEM containing only 10% FBS. After osteogenic differentiation, calcium deposits were detected by staining with Alizarin Red S (Carlo Erba, Val de Reuil, France). The cells were fixed for one hour at room temperature in a 70% ethanol solution. Subsequently, they were washed with distilled water and then coated with a 2% solution of Alizarin Red for 30 min, so the calcium deposits were marked in orange-red.
(2) Adipogenic differentiation: 2 weeks of stimulation in αMEM containing 10% FBS, dexamethasone 1 µM, insulin 10 µg/mL, indomethacin 0.2 mM and 3-isobutyl-methyl-xanthine 0.5 mM. After adipogenic differentiation, the lipid vacuoles accumulated in the cytoplasm were highlighted by Oil Red O (Alfa Aesar) staining. The cells were fixed with a 10% formalin solution for 1 h at room temperature, then washed and stained with Oil Red O.
(3) Chondrogenic differentiation: 3 weeks of stimulation with αMEM containing 1% FBS, 0.1 µM dexamethasone, 6.25 µg/mL insulin, 50 nM ascorbic acid, 10 ng/mL of TGFα. Alcian Blue (Sigma-Aldrich) staining was applied after chondrogenic differentiation, in order to highlight intra- and extracellular glycosaminoglycans. After fixation with a 10% formalin for 1 h, the cells were washed and stained with Alcian Blue for 15 min.

For each differentiation, images were selected for each well (8 composites, 1 negative control and 1 positive control) at 40x magnification.

#### 2.4.2. Viability Test by MTT Assay

The MTT assay (3-[4,5-dimethylthiazol-2-yl]-2,5-diphenyl-tetrazolium bromide) was applied in order to evaluate the cell viability and proliferation rate. This test is based on the intracellular reduction of tetrazolium salts by the mitochondrial enzyme succinate dehydrogenase (SDH) and forming blue-colored formazan crystals, and can therefore only occur in living and metabolically active cells. The value of the optical density (OD) can be correlated to the number of viable cells present. Cell growth and viability with respect to different PVA-based composites were evaluated after 72 h of culture. A 0.3 mL volume solution of MTT (Sigma-Aldrich) was added in each well containing αMEM growth medium and allowed to incubate 3 h at 37 °C with 5% CO_2_. Subsequently, the MTT solution in αMEM was eliminated and replaced with 1.5 mL of isopropanol (Sigma-Aldrich). Quantitative measurement of formazan accumulated in viable cells of the monolayer was performed by reading the OD at 600 nm, using Biophotometer (Eppendorf). The data collected were statistically analyzed by one-way ANOVA (*p* < 0.05).

## 3. Results

### 3.1. Characterization of Se-Doped TiO_2_ Particles

**Figure 1:**
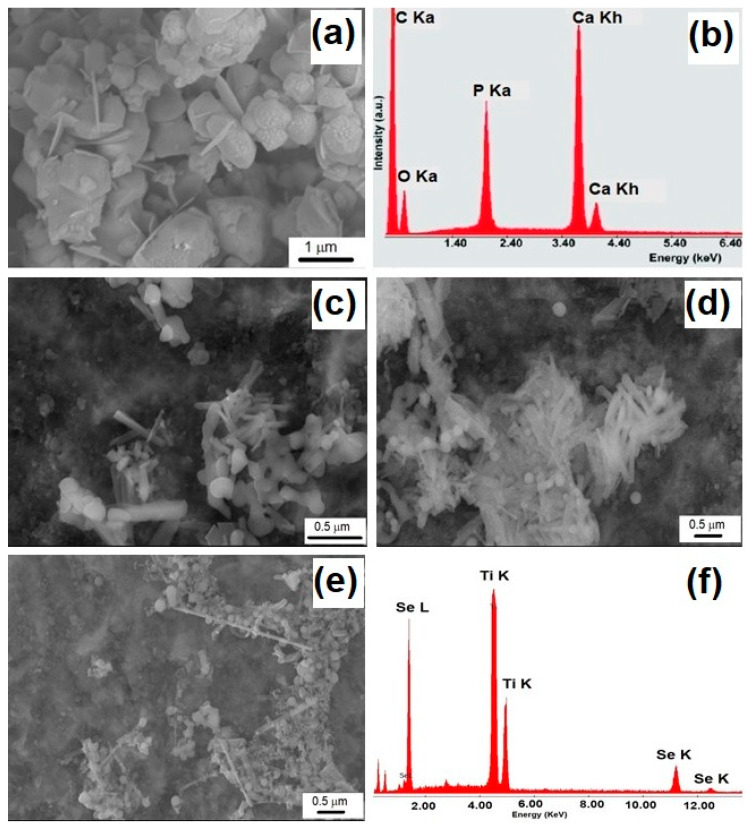
SEM micrographs of hydroxyapatite (HA) crystals (**a**) and Se-doped TiO_2_ nanoparticles obtained from a TiO_2_ precursor calcinated at 400 (**c**), 600 (**d**) and 800 °C (**e**) along with the corresponding EDX spectra (**b**,**f**).

The SEM morphological details of the Se-doped TiO_2_ and HA particles are presented in Figure 1a–f, along with the corresponding EDX spectra. As shown in Figure 1a, the grain size of the HA powder is within microscale, showing a cauliflower shape, while in Figure 1c,d, one can notice the rod-shaped morphology of the TiO_2_ nanoparticles (with the diameter of about 150 nm and variable length) and spherical selenium nanoparticles with a diameter of about 250 nm, intercalated between the TiO_2_ nanorods and also dispersed on their surface. As the calcination temperature of the precursor TiO_2_ particles was increased up to 800 °C, the rod-shaped particles turned to nanowires (Figure 1e) with a smaller diameter (about 80 nm) and spherical selenium nanoparticles with a variable diameter (ranging from 80 nm to 200 nm) randomly distributed and agglomerated around the nanowires.

**Figure 2:**
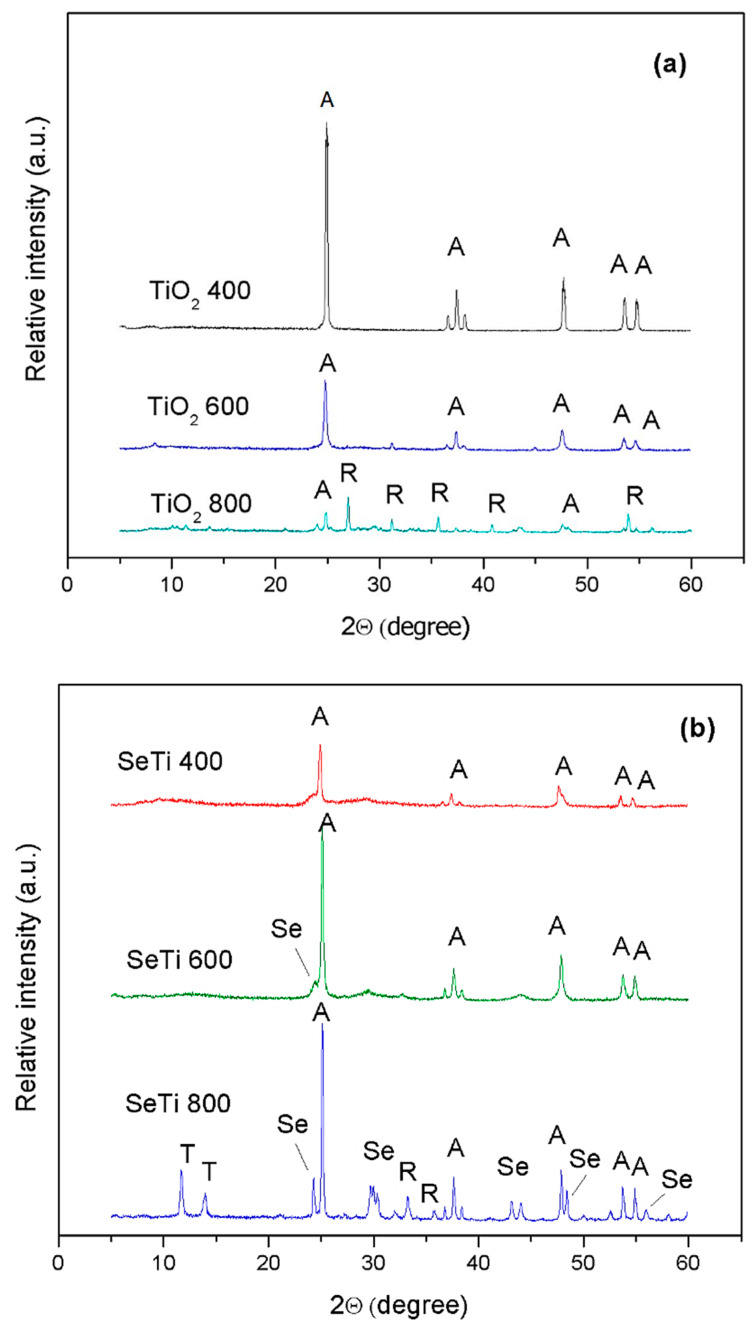
XRD pattern of precursor TiO_2_ particles calcinated at different temperatures (400 °C, 600 °C and 800 °C) (**a**) and corresponding Se-doped TiO_2_ particles (**b**). The symbols are: A—anatase, R—rutile, Se—elemental selenium, T—reduced titanate.

The crystallinity of TiO_2_ and Se-doped TiO_2_ nanoparticles is evidenced by the XRD pattern presented in Figure 2. The evolution of XRD pattern of TiO_2_ particles (Figure 2a) shows the typical crystalline pattern of tetragonal anatase, with sharp peaks at 24.99°, 37.5°, 47.75°, 53.6° and 54.8°, in good agreement with the standard diffraction data corresponding to (101), (112), (200), (105) and (211) crystallographic orientation (PDF-2 Database on CD-ROM, JCPDS—International Centre for Diffraction Data, Newton Square, PA, USA, 2001). As the calcination temperature was increased from 400 °C up to 800 °C, diffraction peaks corresponding to the mixture of anatase and rutile phases, concomitant with the reduction of the anatase peaks’ intensity [34,35], were registered. The XRD pattern of Se-doped TiO_2_ nanoparticles (Figure 2b) showed the same characteristic peaks of anatase, but as the calcination temperature was increased, the peaks became sharper and more intense. Additionally, the patterns corresponding to these nanoparticles show the typical peaks belonging to trigonal Se(0) crystals [36] with the main peaks at 2θ values of 24°, 29.7°, 42.4°, 43.7° and 48.8° corresponding to the crystal planes (100), (101), (110), (102) and (111), respectively, being clearly visible only in the sample calcinated at 800 °C. For the SeTi400 and SeTi600, the shoulder at 2θ = 24° indicates that amorphous nanoselenium particles [37] can convert into trigonal Se phase by phase transition influenced by the temperature of hydrothermal reaction. In the low-degree region, at 2θ = 11.5° and 14°, the pattern of reduced titanate is also visible [38].

### 3.2. Structural and Morphological Characterization of PVA/Se-TiO_2_ and PVA/HA/SeTiO_2_ Composites

**Figure 3:**
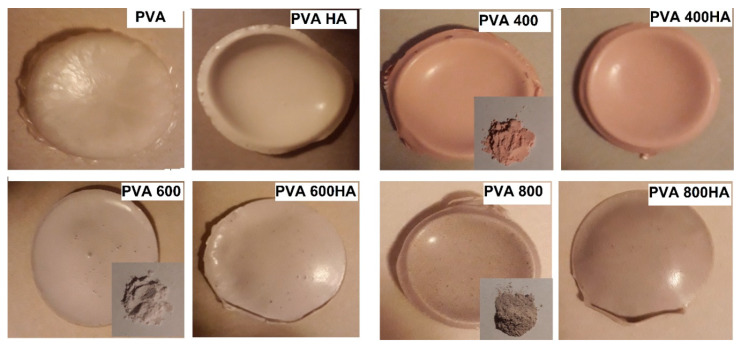
Photographic images of poly(vinyl alcohol)/Se-doped TiO_2_ composites with and without HA and the reference PVA specimen (10%). The inset images present the powder of Se-doped TiO_2_ particles prepared from TiO_2_ precursor after calcination at 400, 600 and 800 °C, respectively.

The composites, as removed from the Petri dishes, after three freezing-thawing cycles applied to the specimens, are presented in Figure 3, along with the photographic image of powder Se-doped TiO_2_ nanoparticles. It can be noticed that the color of the composites changes from pink to gray as the Se nanoparticles undergo a phase transformation from amorphous (pink-red) to trigonal crystalline (dark gray).

#### 3.2.1. FTIR Spectroscopy of PVA Composites

**Figure 4:**
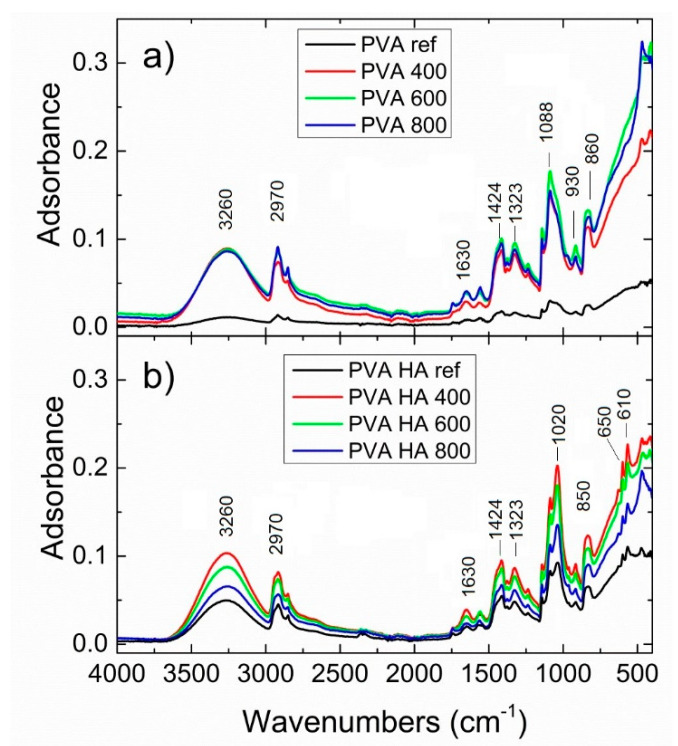
FTIR spectra of PVA/Se-doped TiO_2_ composites (**a**) and PVA/HA/Se-doped TiO_2_ composites (**b**).

Incorporation of Se-doped TiO_2_ and HA particles into the PVA matrix is demonstrated by the FTIR spectra presented in Figure 4a,b, the reference spectrum being PVA 10%. In the high wavenumber region, the typical feature of PVA is represented by the O-H stretching band of both internal hydrogen bonding OH groups or terminal vinyl OH groups, as a broad band centered at 3260 cm^−1^. Two intense peaks at 2910 and 2970 cm^−1^ arising from the C-H stretching of the alkyl groups are assigned to the C-H symmetric and CH_2_ asymmetric stretching vibration, respectively, with their position suggesting that the hydrocarbon chains of the polymer take a trans-zigzag conformation [10,11]. The absorption bands at 1630 cm^−1^ are due to symmetric stretching of the carboxylate anion (-COO-). The band at about 1424 cm^−1^ can be attributed to the -CH_2_ bending and deformation of C-CH_3_, while 1323 cm^−1^ can be associated with CH_2_ wagging, C-C, C-O-C stretching vibrations. The most intense peak in the low wavenumber region is 1088 cm^−1^, assigned to -C-O-H bending and C-O stretching vibration coupled with O-H bending. The shoulder arising at about 1020 cm^−1^ is due to the overlapping with the Se-O bond stretching peaks and, respectively, symmetric stretching vibration. At the same time, the intensity of the Ti-O stretching and Ti-O-Ti bending vibrational modes in anatase are visible at 930 cm^−1^ and 860 cm^−1^ [39]. Upon the HA addition, significant modifications are noticed in the shape and relative intensity of the bands located below 1100 cm^−1^, especially for the PVA400HA sample. The main band is low-shifted at 1020 cm^−1^, as a result of the characteristic vibration mode of P-O bonds in tetrahedral PO_4_^3−^ groups. Concomitant, some additional bands arise at about 610 cm^−1^, assigned to O-P-O bending mode, along with 650 cm^−1^ assigned to Ti-O-Ti stretching mode [40,41]. Overall, the interaction between Se, TiO_2_ nanoparticles and PVA is confirmed from the FTIR features due to the change in the fingerprint regions, as well as the notable changes in the shape and intensities in the region of 3000 up to 3500 cm^−1^ and the significant changes below 1100 cm^−1^. However, it seems that the best interaction between HA particles and PVA hydrogel is observed within the sample PVA400HA.

#### 3.2.2. XRD Patterns of PVA Composites

**Figure 5:**
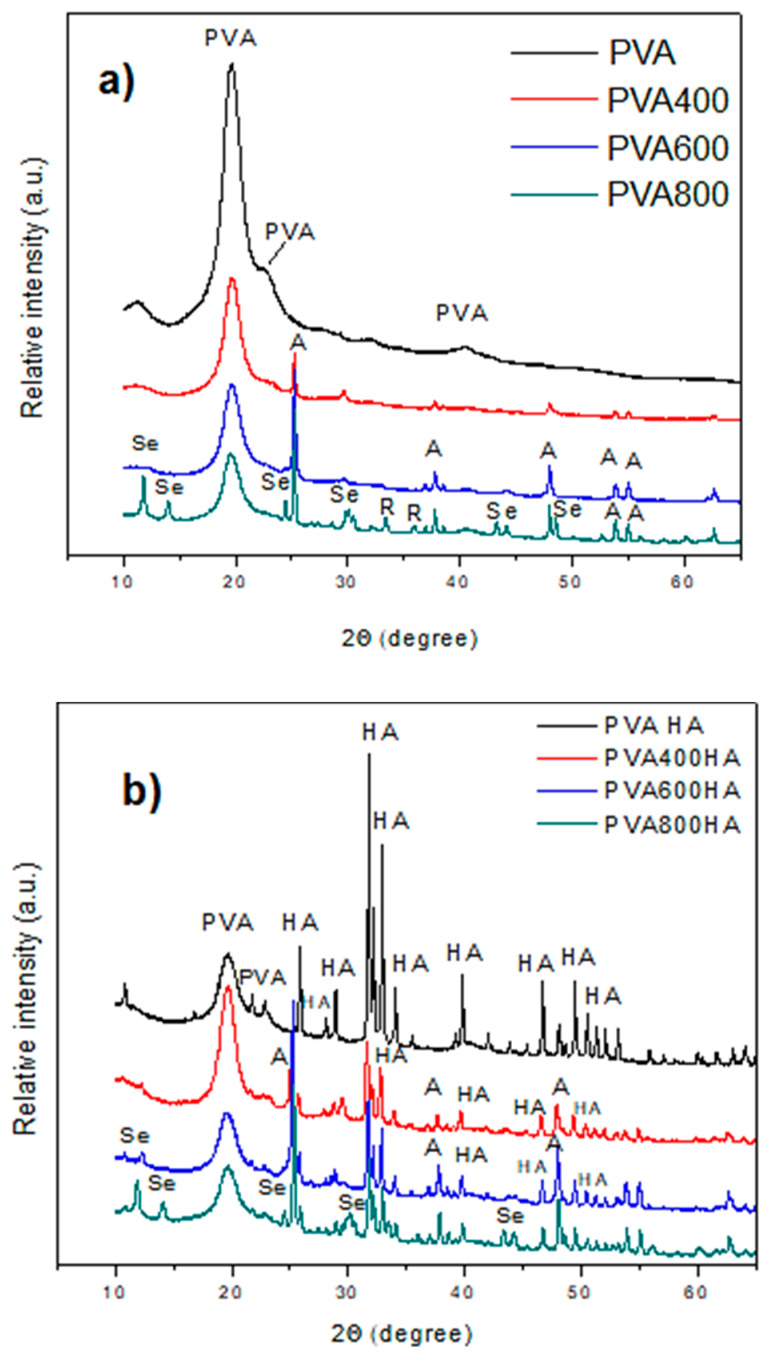
XRD patterns of PVA composites: (**a**) PVA-based composites with Se-doped TiO_2_ particles incorporated; (**b**) PVA-based composites with HA and Se-doped TiO_2_ particles incorporated.

The XRD patterns of pure PVA and PVA composites with and without HA are displayed in Figure 5a,b.

In the pure PVA pattern, the intense characteristic peak at 2θ = 19.8° corresponding to a semi-crystalline structure [42,43]. The presence of the anatase phase of titania nanoparticles is also evidenced at about the same peak location as in Figure 2, along with the weak characteristic peaks of selenium and rutile, mainly emphasized in the samples PVA800. In Figure 5b, the main diffraction peaks at 2θ = 25.90° (002), 31.80° (211), 32.22° (112), 34.08° (202), 39.85° (310), 46.75° (222), 49.51° (213), and 50.54° (321) are assigned to HA (PDF-2 Database on CD-ROM, JCPDS—International Centre for Diffraction Data, Newton Square, PA, USA, 2001). These peaks are well preserved in the XRD spectra of all the composites, but a significant reduction of their intensity can be noticed for the composites PVA400HA, PVA600HA and PVA800HA. This finding demonstrates that HA was well embedded and uniformly dispersed in the PVA matrix, in agreement with SEM characterization.

#### 3.2.3. Morphological Characterization by SEM

**Figure 6:**
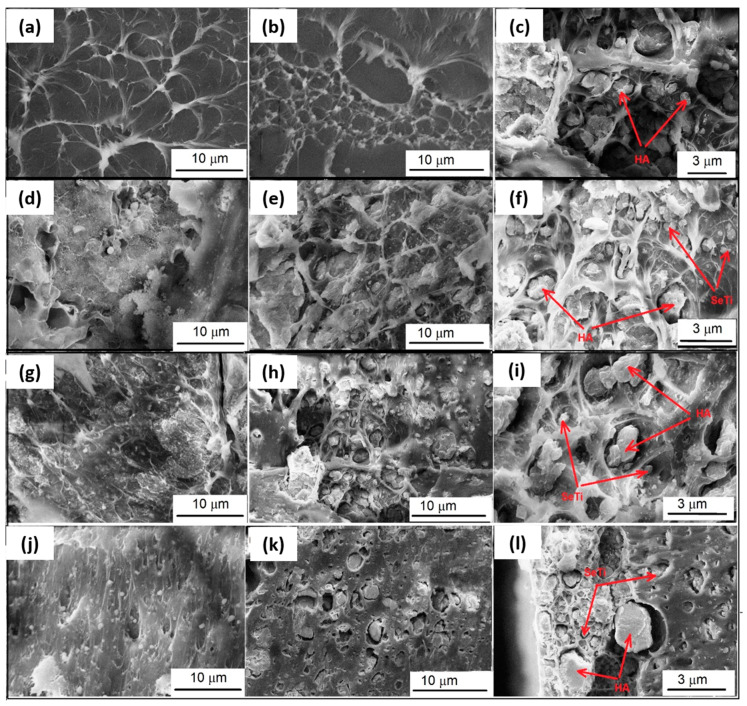
SEM images (cross section) of the PVA composites reinforced with Se-doped TiO_2_ nanoparticles and HA, with different details: (**a**) PVA 10%; (**b**,**c**) PVA HA; (**d**) PVA 400; (**e**,**f**) PVA 400HA; (**g**) PVA 600; (**h**,**i**) PVA 600HA; (**j**) PVA 800; (**k**,**l**) PVA 800HA.

**Figure 7:**
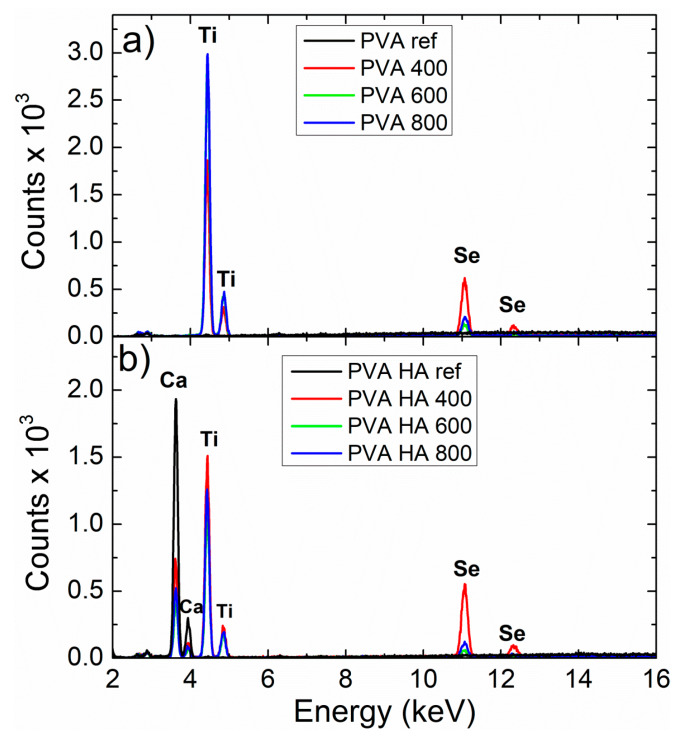
XRF spectra of the PVA specimens: (**a**) PVA/SeTiO_2_ composites; (**b**) PVA/HA/Se–TiO_2_ composites.

Morphological features of the composites at different magnifications are presented in Figure 6, while in Figure 7 the corresponding XRF spectra of the composites with and without HA are displayed. One can observe a “honeycomb” ultrastructure of PVA, filled with HA particles (the large grains) and Se-doped TiO_2_ nanoparticles, randomly dispersed in the polymeric matrix. The morphology of the PVA specimen differs from the composites filled with Se-doped TiO_2_ and HA, in which HA forms fibrous morphology among pores and particles. The observed features indicate that the presence of HA in PVA composites strongly influences the sample morphology, producing a fibrous and porous structure, which may provide advantages in using this material as soft tissue replacement, with interconnected small pores for micro-vascularization. At the same time, the presence of HA inside the “honeycomb” may have an important role in the adhesion ability of the osteogenic and chondrogenic precursor cells, supporting their differentiation, as well as the proliferation [44,45].

### 3.3. Nanoindentation Measurements

**Figure 8:**
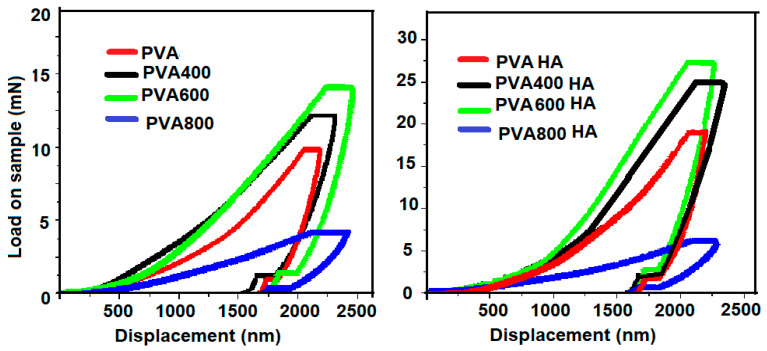
Load–displacement curves recorded for the PVA samples with (**right**) and without (**left**) HA.

Load-displacement curves were recorded for the two sets of PVA samples, with and without HA, respectively, and are presented in Figure 8.

**Figure 9:**
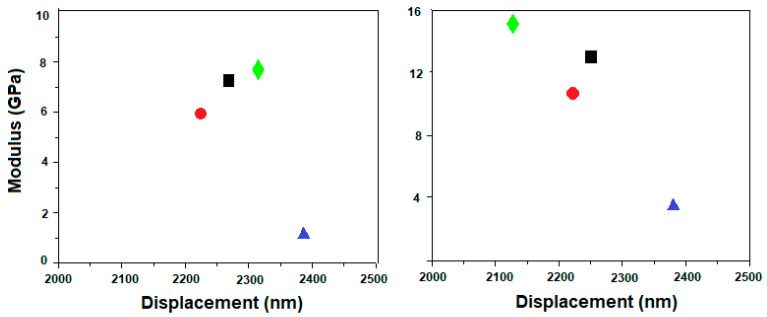
Young modulus calculations (average values) with respect to each set of PVA samples, with (**right panel**) and without (**left panel**) HA. Legend: PVA 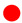; PVA400 and PVA400HA 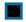; PVA600 and PVA600HA 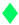; PVA800 and PVA800HA 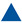.

According to the curves’ profile presented in Figure 8, one can observe that both Se-doped TiO_2_ and HA particles added to the PVA matrix enhance the mechanical properties of the composites, except for the PVA800 sample. A peak force within the 9–15 mN range was necessary to apply on the surface of PVA, PVA400 and PVA600 samples in order to reach the same displacement, while only 4 mN was required for the same indentation in the case of the PVA800 sample. As a comparison, after the HA addition, higher values of peak forces were applied ranging between 17 and 27 mN, in order to reach the same indentation on the surface of the PVAHA, PVA400HA and PVA600HA specimens. The behavior of PVA800HA sample does not correspond to the same pattern, as the maximum force was only 5 mN. Average elastic modulus values obtained from the fitting parameters (Figure 9) revealed a maximum value E = 7.9 GPa for PVA600, compared with E = 6 GPa for PVA as a reference sample. The lowest value was E = 1.6 GPa corresponding to PVA800. The same pattern was observed after the HA addition, as the Young modulus values increased to E = 15 GPa for PVA600HA, while only E = 3 GPa was obtained for PVA800HA. However, as a general behavior, a synergic effect of the Se-doped TiO_2_ and HA particles was noticed in terms of the nanomechanical improvement. 

### 3.4. In Vitro Cells Culture, BMMSC Viability, Differentiation and Proliferation

**Figure 10:**
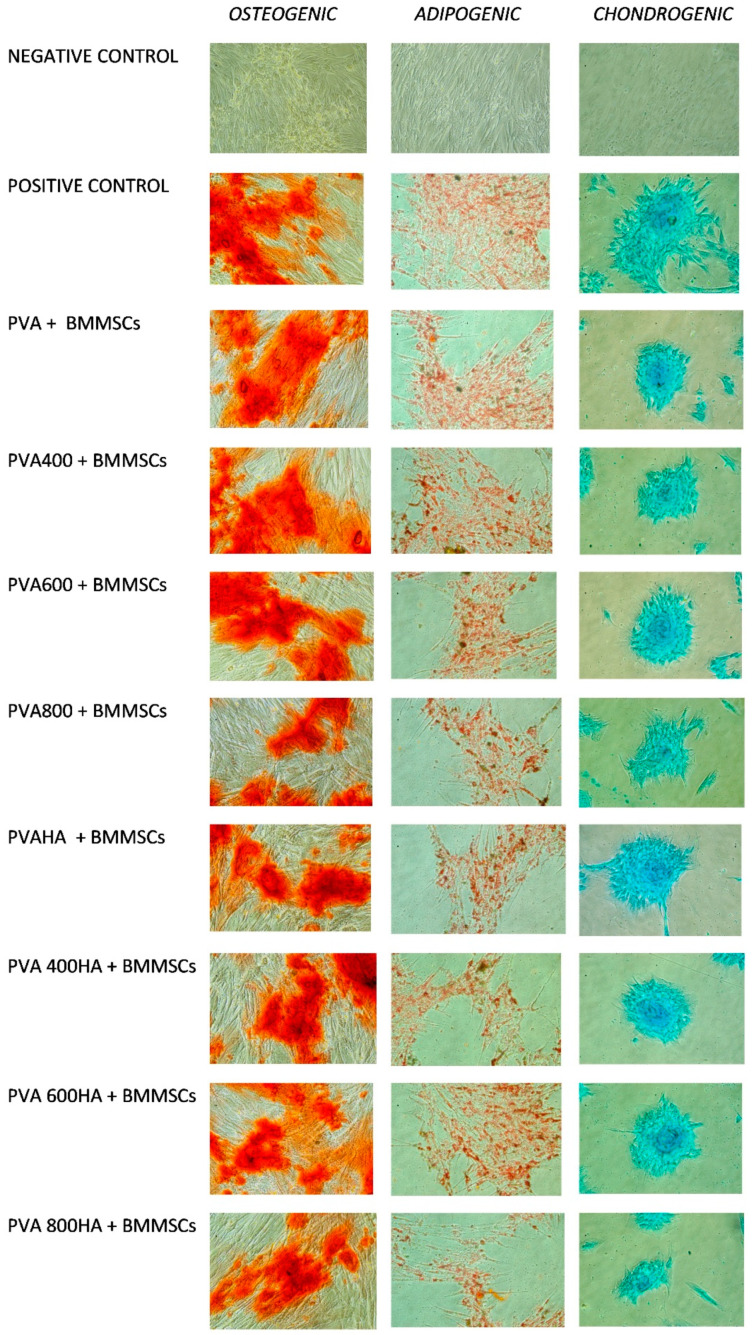
Differentiation potential of bone marrow mesenchymal stem cells (BMMSCs) to adipogenic, osteogenic and chondrogenic lineages, after 72 h incubation time, in the presence of PVA-based composites.

The differentiation potential of BMMSC to adipogenic, osteogenic and chondrogenic lineages is evidenced in Figure 10, along with the positive and negative control. The positive control was obtained by growing the BMMSCs in the differentiating media (osteogenic, adipogenic and chondrogenic), but without the addition of PVA. The negative control was obtained by growing the BMMSCs in MEM (Gibco) with 10% of FBS (fetal bovine serum, Gibco). After specific staining, it is observed that the BMMSCs confirm their differentiation capacity, after specific stimulation, while in the negative control, as expected, the BMMSCs remain undifferentiated. As an overall behavior, it seems that all the composites show better performance for the osteogenic and chondrogenic differentiation rather than for the adipogenic one. BMMSCs had fibroblast-like morphologies, maintaining their normal spindle shape in the early passages, while at later passages, exhibiting less concordant cell morphologies, with some cells characterized by an irregular flattened geometry and enlarged size. Particularly, the chondrogenic stimulation exhibited a distinct extracellular cartilage matrix stained with Alcian Blue (Sigma-Aldrich). At the same time, for osteogenic differentiation, Alizarin (Sigma-Aldrich) staining showed the formation of calcium oxalates on the differentiated BMMSCs, which was not observed in the undifferentiated cells. Intracellular lipid droplets staining using Oil Red-O (Sigma-Aldrich) proved the adipogenesis of BMSCs. Comparing the results obtained for different composites, it seems that PVA800 with or without HA shows the lowest differentiation potential in terms of adipogenic, osteogenic and chondrogenic stimulation.

**Figure 11:**
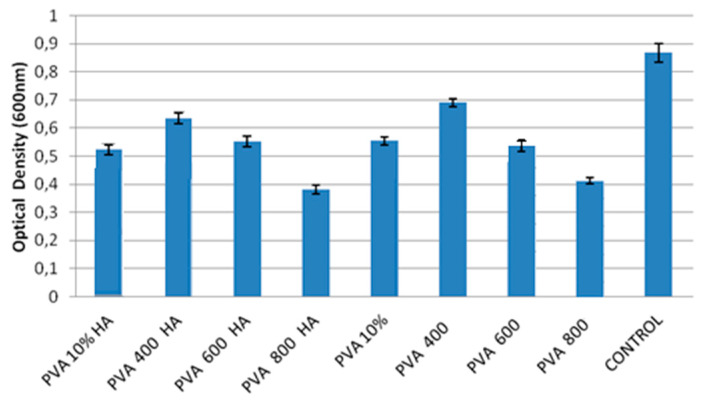
MTT assay of BMMSCs after 72 h incubation with PVA-based composites.

In Figure 11, the MTT assay of BMMSCs after 72 h incubation with PVA-based composites is presented. The best results can be noticed for the PVA400 and PVA400 HA, while the lowest viability was observed for PVA800 and PVA800HA. By comparing with the reference material (PVA 10%), all the composites showed better viability, except the PVA800 and PVA800HA. 

## 4. Discussion

PVA hydrogel is a versatile material, considered to be an ideal alternative for artificial cartilage, meniscus, glenoid labrum (humeral head), but also for trabecular or cortical bone and other orthopedic implants, depending on the targeted applications [1,4,8,45]. It was demonstrated that improved mechanical properties are achieved by physically crosslinked PVA hydrogels toward chemical or irradiative crosslinking techniques [43]. The physical crosslinking technique involves repeated freezing and thawing cycles, forming strong hydrogen bonding among polymer chains and resulting in crystallite formation, which are able to distribute the mechanical loads. The structural and mechanical properties of PVA hydrogel can be manipulated by different variables, such as the number and duration of cycles, polymer content and temperature [43,46,47]. However, the main shortcomings of PVA hydrogel are related to poor strength and bioactivity. To overcome this situation, reinforcing elements, such as different types of nanoparticles and/or HA, are added to the PVA matrix. Among organic nanofillers, CNTs (carbon nanotubes) and graphene appear to be an effective reinforcement for the preparation of PVA nanocomposites [26,48], but they have a drawback related to the high production costs and low dispersion in the polymer matrix. The mineral nanofillers include different clays, metallic nanoparticles (Ag, Au) and metal oxide nanoparticles (TiO_2_, SiO_2_, Al_2_O_3_, ZrO_2_), being developed depending on the final application of the prepared nanocomposite [2,17,20,21,23,24].

In our paper, Se-doped TiO_2_ nanoparticles were used as nanofiller, with the main goal of possible multiple advantages, such as osteogenic, anticancer and antimicrobial properties. It was previously demonstrated that the combination of Se and TiO_2_ nanoparticles display antibacterial, anti-inflammatory properties and inhibit macrophage proliferation with low toxicity to other cells [49] and Se substituted HA nanoparticles shows an antitumor effect on hepatocellular carcinoma [50]. We succeeded in preparing Se-doped TiO_2_ nanoparticles via a hydrothermal reaction, characterized by XRD and SEM, emphasizing their morphology and crystallinity. Depending on the calcination temperature of the TiO_2_ precursor, the morphology of the nanoparticles turns from nanorods to nanowires, while Se nanoparticles preserve their spherical shape. On the other hand, as the calcination temperature was increased from 400 °C up to 800 °C, the appearance of the rutile phase was registered, which is not surprising, as demonstrated by other researchers [34,35]. For example, Zavala et al. studied the influence of preparation temperature, acid treatment and annealing temperature on the structure and morphology of TiO_2_ nanotubes during the formation process and demonstrated the crystallinity modification from anatase to a monoclinic, concomitant with modifications of dimensions, structure and morphology of the nanoparticles. On the other hand, Se can exist in different allotropic forms: amorphous, monoclinic and trigonal phase, the trigonal selenium (t-Se) phase being the most thermodynamically stable [36].

Some authors [51] reported that, from a thermodynamic point of view, the transformation of amorphous Se into crystalline allotropes (either monoclinic or trigonal) requires relatively high temperatures, more precisely, the amorphous form is transformed into the monoclinic Se at around 70 °C. However, some previous studies suggested that amorphous Se forms (or even low crystallinity) are advantageous as they exhibit better solubility and, therefore, higher rates of dissolution and subsequent adsorption and bioavailability [37]. Hence, the amorphous Se nanoformulation is more suitable for drug delivery applications, since nano-Se not only proved a higher efficiency in up-regulating selenoenzymes, but also seem to be less toxic compared to other Se forms [37].

In order to overcome the disadvantage of poor adherence to PVA, hydrogels with both bioactive and biocompatible components were developed by adding HA particles as a second reinforcing phase. The idea is not new, with several studies being reported in the literature [40,44,46,47,52]. For example, Wu et al. [52] have incorporated HA into PVA hydrogel and developed a bioactive composite suitable for orthopedic applications such as artificial cartilage, studying the correlation between the immersion time in SBF (simulated body fluid) and the mechanical properties of these composites. Young modulus was found to be, initially, directly correlated with the immersion time, due to apatite formation on the composite material’s surface, but after prolonged immersion, it remained constant. 

The structural characteristics of PVA and PVA composites at the molecular level were evaluated by the FTIR spectroscopy, which demonstrated the interaction between the PVA matrix, SeTiO_2_ and HA nanoparticles through the changes in position and intensity of peaks occurred in the fingerprint region. XRD patterns of the composites confirm the semi-crystalline structure of PVA along with the crystalline nature of HA, well dispersed in the PVA matrix. The morphology of the composites evidenced a good dispersion of both Se-doped TiO_2_ and HA particles within the polymeric hydrogel, resulting in a reinforced fibrous structure. Se-doped TiO_2_ nanoparticles were deeply embedded in the polymeric network, while the larger HA particles filled the porous architecture. It is obvious that the presence of HA particles influences the porosity of the PVA matrix. Even if the texture of the cross-sectioned area may present some artifacts under the SEM investigation, the details presented in Figure 6 are also influenced by the preparation technique, as the physical cross-linking does not involve a chemical bond between the organic and inorganic phase in the blend. In this case, the ultrastructure of the composites is influenced by the concentration of PVA and number of freeze-thaw cycles, allowing tailoring of the hydrogel network. In a physical cross-linking protocol, during exposure to cold temperatures, water freezes, expelling PVA and forming regions of high PVA concentration. As the PVA chains come into close contact with each other, crystallite formation and hydrogen bonding occur. These interactions remain intact following thawing and develop a three-dimensional hydrogel network. Both hydrogen bonding and crystallite formation are influenced by the number, duration and rate of freeze-thaw cycles. An advantage of using freeze-thaw method is that the toxic chemicals’ involvement during the production can be avoided completely.

Some authors reported that the concentration of HA molecules per composite volume unit is directly correlated to the network stability and to a decrease in the number of pores developed in the structure [47]. In our work, the SEM details clearly demonstrated that HA particles stabilize the fibrous structure, except for the PVA800HA composite. It seems that in this last case, due to the phase transformation of Se and TiO_2_ nanoparticles, the inclusion of HA was not completed. This fact is also supported by the FTIR spectra, which demonstrated that the best interaction between HA particles and the PVA matrix occurred for the sample PVA400HA, in opposition to PVA800HA.

Articular cartilage displays unique morphological and biomechanical properties. Its heterogeneous structure is characterized by a depth-dependent composition and, as a result, it determines nonlinear compression and tension properties and various stiffnesses at different structural levels [53]. PVA hydrogel is a suitable biomaterial from this point of view, due to the possibility to tailor its modulus. The stiffness of PVA can be tailored by selecting the PVA concentration and the number of freeze-thaw cycles, as well as the concentration of reinforcing particles. By means of the nanoindentation technique, we demonstrated synergic improvement of the mechanical properties of PVA by Se/TiO_2_ and HA reinforcement. Based on the load–displacement curves and Young modulus calculations, we found that the highest stiffness was reached for the PVA600HA composition (E = 15 GPa), while PVA800HA was the softest one (E = 3 GPa). It was expected that the addition of inorganic particles, especially hydroxyapatite, leads to the improvement in the mechanical strength of PVA, correlated to the surface energy interactions generated between the matrix and the reinforcement, as already demonstrated by previous studies [40,46,47]. As an interesting comparison, Ferrara et al. [54] applied nanomechanical testing methods on jaw cartilage of sharks which possesses a unique arrangement of both mineralized and soft layers, as an intermediate structure between bone and cartilage. At a peak force of 1000 μN, they found values of Young modulus ranging from 2 GPa to 4 GPa for the mineralized cartilage, while for the non-mineralized one, the values were much lower (E = 20–40 MPa). However, different values of Young modulus have been reported for human articular cartilage and meniscus, depending on several factors such as strength, indentation depth, dehydration degree and anatomical position [9,53,55]. According to a critical review [56], indentation hardness measurements at macro-, micro- and nanoscale differ in a couple of ways and the results are expected to be comparable if tip geometries are used providing similar strains in the tested material, such as the Vickers and Berkovich shaped tips. These differences also concern the comparison between the size of the indentation surface and the size of the heterogeneities of the material. During the nanoindentation test, the displacement and the load are continuously monitored with high precision, the indenter will penetrate the sample until a predetermined maximum load is reached, where the corresponding penetration depth is at maximum value. The local properties measured are linked with the size of the contact area, therefore of the homogenized properties under the contact, which are very different at the macro-, micro- or nanoscale. Specifically, in nanoindentation tests, the contact area is calculated according to Oliver-Pharr equations [31] and not directly measured, and hence, at maximum load, the contact area may increase and the measured elastic modulus and hardness can be significantly overestimated. On the other hand, when comparing different indentation hardness, the heterogeneous architecture of articular cartilage has to be considered, with a depth-dependent composition and structure, showing anisotropic and nonlinear properties in compression and tension [53].

Highly tailorable properties of PVA-based composites and their potential use in regenerative therapies and tissue engineering can be assessed by BMSCs differentiation toward different cell lineages, since they can be isolated from different tissues and cultured in vitro. It is well known that biomaterials can induce the MSCs differentiation by modulating features of the substrate such as the composition, topography and mechanical properties [57]. Particularly, materials with increased stiffness can induce and activate osteoinductive growth factors, while different types of hydrogels (PVA, PEG, alginate) enabled BMMSCs chondrogenesis and cartilaginous matrix formation both in vitro and in vivo [58]. In a recent study, Ahmed et al. [59] clarify that Se/Ti nanocomposites possess a proliferative potential with respect to MSCs, highlighting the influence of nanocomposite texture on the enhancement of the BMMSCs proliferation, owing to the increase of titanium concentration. The authors’ motivation was to stabilize the Se through complexation with titanium in order to maximize its biological effect toward BMMSCs proliferation. In the present work, we demonstrated that PVA/SeTiO_2_ and PVA/SeTiO_2_/HA composites show better performance for the osteogenic and chondrogenic differentiation than for the adipogenic one. The differentiation potential of the BMMSC cells toward the PVA800 and PVA800HA composites was less evidenced, probably influenced by the phase transformation of Se and TiO_2_ nanoparticles, and, consequently, the microstructure and surface topography of the composite. This result was also supported by the MTT viability test. The best viability was noticed for the PVA400 and PVA400HA compositions. 

It is well known that the main drawback of using nanoparticles as filler material for polymeric biomaterials is related to the toxic response, due to the wear debris from artificial joints, as they are able to enter the body via the circulatory and lymphatic system. The potential adverse effects may range from inflammation to fibrosis and carcinogenesis [60]. Therefore, the interactions between nanomaterials and surrounding tissues, as well as their biological effects, are important features to be studied not only in static conditions, but also in vivo, using animal models. Hence, further in vivo investigations are taken into account from this point of view. Within the limitations of our study, in this case, the concentration of Se-doped TiO_2_ particles released from the PVA matrix did not show any toxic effect toward BMMSCs, which is in correlation with previously reported data [59,61,62,63]. Moreover, Perla et al. [63] demonstrated that Se nanoparticles have shown an inhibitory effect on the growth of many cancerous cell lines, while demonstrating a positive effect on osteoblasts growth. Selenium, in the form of nanoparticles, has been found to have low toxicity toward healthy, noncancerous cells, such as osteoblasts and human dermal fibroblasts at a maximum chronic dose of 2400 µg/day [60]. However, at high concentrations, selenium compounds become pro-oxidants, reacting with thiol groups to produce superoxide anions and hydrogen peroxide. In the present study, careful consideration of the selenium salt concentration used in the preparation protocol was made based on our previous research and other related papers in the literature devoted to Se nanoparticle production and possible applications in biomedicine and food science [28,37,49,50,59,64]. For example, Ahmed et al. [59] demonstrated that SeO_2_/TiO_2_ nanocomposites prepared in different ratios (1/4, 1/6, 1/8), possess proliferative potential on MSCs with the optimum effect exerted by the composites with 1/6 ratio Se/TiO_2_ [59]. In the present study, even if the concentration of Se ions in the culture media was not determined, the MTT assay of BMMSCs after 72 h incubation does not show toxicity effect when compared with the control. Compared to the reference PVA, all the composites showed better viability, except the PVA800 and PVA800HA.

Our results also match with some previously reported data in which selenium substituted hydroxyapatite supports the growth of bone marrow stem cells [61], while nanoscale modification of titanium substrates increases proliferation and the formation of the extracellular matrix in the human BMMSCs [62].

## 5. Conclusions

In the present paper, novel hybrid PVA composites were fabricated with the aim of synergic effect in terms of enhanced biocompatibility and mechanical properties. Se-doped TiO_2_ nanoparticles were fabricated by hydrothermal reaction and characterized from a structural and morphological point of view, prior to mixing with the PVA matrix. The PVA/Se-doped TiO_2_ composites with and without HA were structurally investigated by the FTIR and XRD, in order to confirm the incorporation of the inorganic phase in the polymeric structure, and by the SEM and XRF, to evidence the ultrastructural details and dispersion of nanoparticles in the PVA matrix. Both the mechanical and structural properties of the composites demonstrated a synergic reinforcing effect due to HA and Se-doped TiO_2_ nanoparticles. Moreover, the tailorable properties of PVA-based composites were proved by the differentiation potential of the BMMSCs to osteogenic, chondrogenic and adipogenic lineages. The novel hybrid PVA composites show suitable structural, mechanical and biological features to be considered as a promising biomaterial for articular cartilage and subchondral bone repair. By corroborating the structural, mechanical and biological tests, we can remark that the best results were obtained for the PVA600HA and PVA400HA compositions.
